# Are Recycling People Also Saving? Costliness Matters

**DOI:** 10.3389/fpsyg.2020.609371

**Published:** 2021-01-13

**Authors:** Sheng Wei, Jiaqi Xu, Shengxiang She, Yan Wang, Ying Zhang

**Affiliations:** ^1^School of Management, Harbin University of Commerce, Harbin, China; ^2^Center for Behavior and Decision, Shannxi University of Technology, Hanzhong, China; ^3^School of Business, Guizhou University of Finance and Economics, Guiyang, China; ^4^School of Sociology and Psychology, Central University of Finance and Economics, Beijing, China

**Keywords:** recycling effort, resource saving, self-identity in environment, pride feeling, recycling cost

## Abstract

In view of the fact that vigorously promoting recycling has become a viable means to promote sustainable development, it is important to better understand the impact of recycling efforts on subsequent resource saving behavior. This research empirically examines the effects of recycling efforts on subsequent resource saving by analyzing the survey data of 356 college students in China. The recycling efforts, environmental self-identity and feeling of pride were measured using existing scales while saving behaviors and recycling cost were measured by developing new scales. Partial least squares structural equation modeling was performed to test the structural relationships among recycling efforts, environmental self-identity, feeling of pride, and saving behaviors. Further, the moderation role of recycling cost was tested. The results showed that (1) saving behaviors could be classified into two types based on their costliness; (2) recycling efforts have a positive effect on costless saving behaviors, while having a negative effect on costly saving behaviors; (3) both the positive and negative effect of recycling efforts on resource saving is mediated by pride feeling and environmental self-identity; and (4) recycling cost negatively moderates the effects of recycling efforts on pride feeling. We discuss the theoretical and managerial implications of the findings.

## Introduction

China having the highest population in the world not surprisingly produces one of the largest if not the largest amounts of solid waste. Promoting the reduction of solid waste sources and recycling to minimizes the negative impact on the environment is the advocated green urban development model. Recycling is “the process of collecting and processing materials that would otherwise be thrown away as trash and turning them into new products”^1^. Governments and environmental public welfare organizations have invested many resources in cultivating, publicizing and promoting people’s recycling behavior, such as recycling facilities installed in public places, household waste classification and recycling policies, school education program and ubiquitous public service commercials, and propagandas. Many businesses have also launched second-hand goods recycling deduction plans (such as old clothes recycling plan by H&M) and idle goods circulation market (such as Taobao online idle goods market).

Resource recycling is an important mean to achieve sustainable development. However, the main focus on the work so far has been on factors affecting recycling behavior (e.g., Tonglet et al., 2004; Schultz et al., 2007; Goldstein et al., 2008; Trudel et al., 2016; Wang et al., 2016; Echegaray and Hansstein, 2017; Wan et al., 2017). These studies focused on garbage charges, identity exposure, information presentation, commodity appearance, individual attitudes, level of education, and other factors related to recycling habits. The insights from the these studies can help policymakers educate and persuade the public to participate in recycling activities, though the long term overall effect of these efforts is questionable. The basic assumption underlying such research is that garbage recycling is an effective way to prevent pollution, save energy, and save natural resources (Troschinetz and Mihelcic, 2009; Varotto and Spagnolli, 2017). Nevertheless, an initial sustainable act may lead the individual to perform unsustainable behaviors (Meijers et al., 2013), so peoples’ recycling efforts may increase their levels of future consumption, thus increasing overall resource use. For example, consumers who recycled their used clothes may feel that buying new clothes is more acceptable. If the potential negative consequences of recycling (such as promoting waste) cannot be avoided, it will be hard for policymakers to maximize the beneficial effects of recycling (Ma et al., 2019). Therefore, it is very important to study the subsequent resource saving behavior related to involvement in waste recycling.

The aftermath of recycling behavior has received limited investigation and the questions whether it encourages saving or wasteful behavior remains to be answered. Social psychology studies suggest that humans have motivation for consistent behaviors (Beaman et al., 1983; Burger, 1999; Mullen and Monin, 2016). The theory of self-perception suggests that people will infer their attitudes, beliefs and self-characterizations according to their previous behavior, and then make choices consistent with self-concept. Past environment sustainable behavior leads to the perception that “I am a pro-environmental person,” and this perception promotes the subsequent emergence of similar behavior (Bénabou and Tirole, 2011). Studies have found that moral behavior promotes subsequent moral behavior (Gneezy et al., 2012), and green consumption promotes green consumption or environmental behavior (Cornelissen et al., 2013; Summers et al., 2016). On the other hand, a large amount of literature illustrated just the opposite, i.e., when individuals took sustainable behavior before, they will reduce their sustainable behavior or engage in unsustainable behavior subsequently. For example, after consuming green products, the possibility of purchasing the green products in the future decreases (Mazar and Zhong, 2010). Compared with ordinary cars, hybrid cars drivers use cars more frequently, drive more mileage (Sun and Trudel, 2017), and have more traffic violations and accidents (Woodyard, 2009). Individual support for green funds is lower after garbage recycling (Truelove et al., 2016). When economic incentives were used to encourage household waste recycling, the consumption of electricity in the same household increased (Xu et al., 2018).

A recent study on how recycling behavior affects future consumption comes from Ma et al. (2019) who investigated the negative consequences of recycling behavior. Although previous studies have given conflicting conclusions, their results provide evidence to support the prediction that recycling activities indeed increase future resource consumption. This effect is mediated by two mechanisms, i.e., pride feeling and environmental self-identity, that decrease negative emotions from wasting behaviors. Feelings of pride, as a self-conscious emotion, play an important role in self-regulation. Environmental self-identity, the degree to which individuals regard themselves as environmentalists (Whitmarsh and O’Neill, 2010; Werff et al., 2013, 2014) were found to decrease negative self-attributions associated with wasteful consumption (Bolton et al., 2006; Ma et al., 2019). Furthermore, they indicated that the consideration of future consequences negatively moderates the effects of recycling efforts on pride feeling and environmental self-identity. Based on the above evidence, we tend to presume that individuals’ recycling efforts would reduce their saving behavior. Unfortunately, few studies have examined the relationship between recycling efforts and resource saving behavior. Existing studies have either only focused a specific consumption behavior (such as using paper cups or scrap paper) in an experimental research situation (Catlin and Wang, 2013; Sun and Trudel, 2017) or taken a proxy variable of resource saving, such as average monthly expenditure (Ma et al., 2019), which provided limited insights into the understanding of resource saving behaviors.

In this research, we empirically investigate these issues based on survey data from college students in China, using structural equation modeling. We argue that resource saving behavior is a set of behaviors rather than a single action and saving behaviors could be classified into two types, i.e., costly and costless saving behaviors. From a viewpoint of evolution, the perceptions of the costs of saving behaviors might play a substantial role for different individuals (Poškus, 2017). It is suggested that the self-oriented attitude reflecting personal gain may have different effects on environmental behavior than social-oriented attitude reflecting altruism. Although individuals sometimes show socially desirable behaviors such as carrying a reusable shopping bag and try to improve their social status at the cost of losing resources (costly signaling), at other times, they may be reluctant to pay a cost to save resources, so as to maintain their personal advantages (Gintis et al., 2001; McAndrew, 2002; Millet and Dewitte, 2007; Bereczkei et al., 2010). Therefore, the individual’s resource saving behaviors may not only be affected by the previous recycling behavior in terms of moral self-regulation, but also the costliness of recycling behavior can not be ignored. The current study’s framework are based on the Ma et al. (2019)’s study, and further extends it by examining two types of saving behaviors, which shows different consequences. In addition, we investigated the role of recycling cost when examining the effect of recycling efforts on pride feeling and environmental self-identity.

## Hypothesis

To investigate the relationship between recycling efforts and subsequent saving behavior, we used Ma et al. (2019) conceptual framework with modification of adding recycling costliness as a moderator. Furthermore, we used a set of saving behaviors to replace the single indicator (i.e., monthly living expenses) used by Ma et al. (2019), and found that the structure of saving behavior show two dimensions. Ma et al. (2019) proposed that recycling efforts can increase peoples’ consumption level, which is mediated by feelings of pride and environmental self-identity that can reduce negative emotions from resource wasteful behaviors. Firstly, as the study is based on the Ma et al. (2019) framework, we wanted to confirm the relationships observed in the original study and explore the relationships between recycling efforts, environmental self-identity, and pride feeling.

**H1. Recycling efforts positively affect environmental self-identity.**

**H2. Recycling efforts positively affect pride feeling.**

**H3. Pride feeling positively affect environmental self-identity.**

Further, we predict that the impact of recycling efforts on saving behavior depends on the costliness of saving behavior, that is, whether it is costly or costliness for individuals. Recycling efforts promote costless saving behavior, but they inhibit costly saving behavior, which is mediated by pride feeling, and environmental self-identity. In addition, we predict that the cost of recycling activities will moderate the effects of recycling efforts. Below, we discuss each concept in more detail and present our hypotheses.

The findings from empirical research consistently suggested that there is a positive correlation between ecological affect referring to the degree of emotionality an individual is attached to environmental issues and ecological behavior (e.g., green purchase; Chan and Lau, 2000). He et al. (2013) found that ecological emotion (such as pride, cherishing) played a mediating role in the positive impact of green cognition (i.e., knowledge about green products) on green behavior. Sun and Trudel (2017) argued negative emotions experienced during resource wasting behavior can be reduced by positive emotions arousing from the following recycling or resource saving activities. Thus, pride feelings are likely to decrease resource saving by reducing the negative emotions of wasteful behavior (Ma et al., 2019). Besides, based on social exchange theory’s rank equilibrium norm (Cropanzano and Mitchell, 2005), individuals’ feeling of pride arousing from their recycling efforts, makes them feel more entitled to make less other environmentally responsible decisions, such as feel comfortable about using more resources (Ma et al., 2019). Therefore, we propose:

**H4. Pride feeling negatively affect saving behaviors.**

Previous work on social cognition based on the concept that individuals have different identities (Reed, 2002; DeMarree et al., 2005) has proved that individuals’ prior behaviors can stimulate a certain self-concept and influence their subsequent behaviors. The moral licensing effect showed that previous moral behavior might activate and promote a positive self-concept, thus allowing consumers to make more of self-indulgent choices later (Khan and Dhar, 2006; Merritt et al., 2010). Following this, an increased feeling of environmental self-identity could act as one of the factors for decreasing negative emotions from wasteful behavior (Bolton and Alba, 2012; Sun and Trudel, 2017). Empirical research in the effect of moral licensing found similar evidence. For example, people who see themselves as typical recyclers are more likely to recycle than those who do not perceive themselves as recyclers (Mannetti et al., 2004), garbage recycling leads to lower green fund support (Truelove et al., 2016). In this research context, recycling efforts also have the potential to activate and confirm environmental self-identity, which can be a “get out of jail free card,” making high levels of consumption more acceptable (Whitmarsh and O’Neill, 2010; Ma et al., 2019). Therefore, engaging in recycling could boost environmental self-identity, which decreased the negative self-attribution associated with wasteful consumption (Ma et al., 2019) and, thus decreased the likelihood of saving behaviors.

**H5. Environmental self-identity negatively affects saving behaviors.**

Recycling activity is a collection of multiple actions rather than a single behavior, many of which require individuals to accept some costs, including financial costs (such as purchasing recycling equipment), physical costs (going to a specific recycling point), and mental burdens (garbage sorting), etc. Sun and Trudel (2017). Gneezy et al. (2012) proposed that the costliness of initial pro-social behavior is a key moderator of moral consistency between a sequence of behaviors. Costly pro-social behavior signals a pro-social identity, leading to moral consistency of sequential behaviors, whereas costless behavior does not, leading to no moral consistency. In their experiment, compared to the control condition, participants lied significantly less in the costly condition and significantly more in the costless condition. Participants in the costly condition also rated themselves as more helpful and less selfish than participants in either of the other two conditions, and the difference in truth-telling between the costly and the costless conditions was mediated by this self-rating of pro-social identity.

Accordingly, costly recycling behavior is more diagnostic about oneself, leading people to embrace the value indicated by that behavior (Burger, 1999; Gneezy et al., 2012). Individuals who accept the higher cost of recycling, which can prove that they are a person who abides by social norms, thus strengthening their environmental identity. In addition, if the recycling was costly, it means that consumers contributed more efforts for recycling, which will be converted into more moral credits or improve the quality of moral credentials (a mechanism of moral licensing). In the model built by Sun and Trudel (2017), higher recycling cost (for example, putting the recycling bin farther away in the field experiment) would produce stronger positive emotions. Thus, we hypothesize the following:

**H6. Recycling cost positively moderates the effect of recycling efforts on environmental self-identity.**

**H7. Recycling cost positively moderates the effect of recycling efforts on pride feeling.**

**FIGURE 1:**
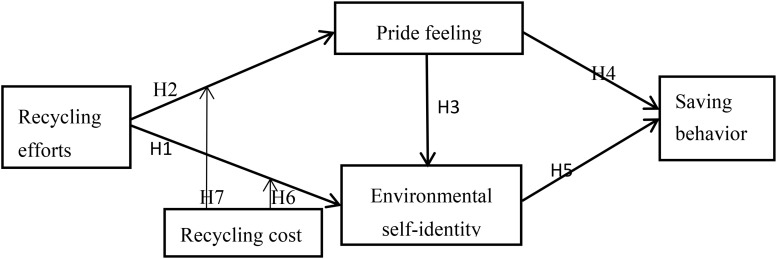
Conceptual model.

We constructed a conceptual model to better present the relationships between each constructs (Figure 1).

## Empirical Study

The subjects of this study were college students majoring in economics and management from Shaanxi University of Technology in China. Respondents who completed the questionnaire were rewarded with money of 10 RMB. A total of 442 questionnaires were sent out during the class and 436 valid sample was obtained. Among them, there were 135 males and 301 females. 58% of the population grew up in rural areas and 42% in urban areas. The *M*_*age*_ was 19.13 (SD = 1.02). Though the females are much more than males in our sample, we included control variables for gender (male/female), and growing background (rural/city), to capture unobservable differences.

We adopt PLS-SEM (partial least squares structural equation modeling) for our data analysis. Using PLS is appropriate because it does not assume normal distributions and allows for analyses with small samples (Hair et al., 2011). Besides, SmartPLS 3.0 can provide consistent results when all constructs are reflective (Dijkstra and Henseler, 2015). If properly used, PLS-SEM can provide more robust estimations of structural models than can covariance-based SEM (Reinartz et al., 2009). Using SmartPLS 3.0 software, a two-stage analytical procedure was applied to analyze the data (Hair et al., 2011). Firstly, we assessed the reliability and validity of the measurement model and then examined the parameters and the explanatory power of the structural model. The significance of the model estimates was based on a bootstrapping procedure with 5,000 samples.

### Measure

The questionnaire included five constructs: recycling efforts, environmental self-identity, pride feeling, saving behaviors, and recycling cost. We used scales from prior research to measure the first three constructs (Ma et al., 2019; See Table 1) and developed the scales to measure saving behaviors and recycling costs.

**TABLE 1:** Constructs and measure.

Construct	To what extent do you agree with the following statements? 1 = strongly disagree, 7 = strongly agree.	Factor loading
Recycling efforts	I usually separate and dispose of all recyclable materials	0.850
	I have high involvement in recycling activities.	0.842
	I tend to buy products which can be recycled in the future.	0.767
	I have high adherence levels to separating and disposing of recyclable materials.	0.839
Environmental self-identity	I think of myself as an environmentally-friendly individual.	0.853
	I think of myself as someone who is very concerned with environmental issues.	0.850
	I would be happy to be seen as having an environmentally-friendly lifestyle	0.829
	I would want my family and friends to think of me as someone who is concerned about environmental issues.	0.767
	I think everyone should contribute to environmental protection.	0.695
Pride feelings	I am proud of my recycling efforts.	0.955
	I feel good about my recycling efforts.	0.959

#### Recycling Efforts, Environmental Self-Identity and Pride Feeling

Recycling efforts was adapted from Ramayah et al. (2012) and Wang et al. (2016), environmental self-identity was adapted from Truelove et al. (2016), and pride feeling was adapted from Harth et al. (2013). The three constructs as well as the measurement also had been used in Chinese consumer context by Ma et al. (2019). To evaluate the psychometric adequacy of the constructs in this study, confirmatory factor analysis were conducted. The factor loadings for each construct are shown in Table 1. All factor loadings are significant (*p* < 0.001), ranging from 0.695 to 0.955. According to Table 2, Cronbach’s alphas for the main constructs are 0.843 or above, and composite reliability ranges from 0.895 to 0.956 (Table 2), both of which exceed the benchmark of 0.7, suggesting that all of these measures are reliable. Convergent validity was assessed using the average variance extracted (AVE) from the constructs. All constructs range from 0.641 to 0.915 in AVE, well above the recommended value of 0.50. According to Fornell and Larcker (1981), the AVE of each construct exceeds its squared correlation to any other construct, assuring the discriminant validity of the constructs. Further, the variance inflation factors of all constructs are lower than the recommended value of 5 (the maximum is 3.228), demonstrating that multicollinearity is not a threat in this research.

**TABLE 2:** Description statistics and reliability of measures.

Measure	Mean	SD	α	AVE	CR	1	2	3
1.Recycling efforts	4.405	1.477	0.843	0.681	0.895	0.825		
2.Environmental self-identity	6.165	0.884	0.860	0.641	0.899	0.368	0.801	
3.Pride feeling	5.917	1.143	0.908	0.915	0.956	0.396	0.559	0.957

*Note: SD, standard deviation; AVE, average variance extracted; and CR, composite reliability.*

#### Saving Behavior and Recycling Cost

This study developed a pool of items to measure saving behavior and recycling costliness since there are no existing scales to use. We tested the initial item pool in qualitative interviews with 20 undergraduate students at a university in Guiyang, China. The final survey instrument was developed by selecting and modifying the items according to feedback from the interviews. The items are shown in the Tables 3, 4, respectively. Prior to reply, the respondents read: to what extent are the following statements consistent with your actual situation? The main constructs were measured with a 7-point Likert scale (1 = very inconsistent, 7 = very consistent). To ensure the validity of the scale, exploratory factor analysis (EFA) was carried out on the two newly designed measures, namely, saving behavior and recycling cost.

**TABLE 3:** Results of exploratory factor analysis of saving behaviour.

item	Factor loading	
	Costless saving behavior	Costly saving behavior
Retain printed papers with a clean side	**0.754**	−0.107
Turn off the lights after use	**0.680**	−0.135
Shut off water while applying bath foam.	**0.676**	−0.121
Use toothpaste to completely squeeze out	**0.661**	0.165
Take shopping bags with you	−0.104	**0.796**
Wear worn-out socks	−0.015	**0.757**
Cronbach *a* coefficient	0.650	0.603
KMO	0.675	
Bartlett spherical test	0.000	

*Note: The items with bold value in a column belong to one factor.*

**TABLE 4:** Exploratory factor analysis of recycling cost.

Item *M* = 4.281 SD = 1.507	Factor loading
I spend time on recycling.	0.818
I paid mental efforts for recycling	0.789
I paid extra money for recycling.	0.714
Recycling is easy for me (Reverse scoring)	0.544
Cronbach *a* coefficient	0.681
KMO	0.650
Bartlett spherical test	0.000

##### Measure of saving behavior

Exploratory factor analysis extracted two factors and explained 45.563% of the total variance. According to the semantic content of measurement items, they are named as costless saving behavior (including 4 items) and costly saving behavior (including two items; see Table 3).

In terms of costliness, there are differences in the difficulty of people’s saving behaviors. Costless saving behaviors are easy to implement, which require not much physical, mental or psychological cost, such as turning off the lights after use. On the contrary, the costly saving behavior is not as easy to take, because it will bring a certain physical or psychological cost. For examples, carrying a shopping bag not only brings trouble but may also appears strange especially for young people. Although wearing worn-out socks is a effort of saving resources, it may detract from one’s self-concept and therefore produce a psychological cost. In fact, the sample on the whole scored higher on costless saving behavior (*M* = 6.22, SD = 1.218) and lower on costly saving behavior (*M* = 3.982, SD = 1.997), indicating that there are substantial differences between the two dimensions of saving behavior for the college students. Considering this fact, it is necessary to distinguish the two types of saving behavior when testing the hypotheses.

##### Measure of recycling cost

As shown in Table 4, EFA extracted one factor and explained 52.466% of the total variance. The factor loads of all items ranged from 0.544 to 0.818, which was greater than the recommended value of 0.4. Cronbach *a* coefficient is 0.681, which is greater than the recommended value of 0.6. Drawing on Sun and Trudel (2017), recycling efforts is associated with financial cost (e.g., purchasing of expensive recycling equipment or recycling depot fees), physical cost (e.g., walking some distance to recycle), and mental cost (e.g., sorting trash and using multiple bins). In our scale, recycling cost mainly covers the time, energy and money paid by individuals in implementing recycling behavior. The fourth item measures the difficulty of recycling as a whole.

## Hypotheses Testing

### Structural Model

Since two different factors are obtained with regard to saving behavior, the influence paths between variables are tested by estimating structural equation models for two kinds of saving behavior, respectively. *R*^2^ level and significance of the path coefficients were used as the primary evaluation criteria for the structural model (Hair et al., 2011). The analysis started by investigation whether the results of the Ma et al. (2019) study still holds when introduced outcome variables of costless saving behavior and costly saving behavior.

#### Costless Saving Behavior as a Dependent Variable

Taking the costless saving behavior as the dependent variable of the structural equation model In H1, following the Ma et al. (2019) study, we tested and evidenced that recycling efforts positively affect environmental self-identity (*b* = 0.174, *t* = 4.051, and *p* < 0.001). In addition, environmental self-identity significantly positively affects costless saving behavior, reflecting a effect of reinforcement, so H5 is rejected (*b* = 0.425, *t* = 6.800, and *p* < 0.05). The path coefficients show that the path between recycling efforts and pride feeling is positive and significant (H2; *b* = 0.396, *t* = 9.434, and *p* < 0.001). The path coefficient indicates that the effect of pride feeling help to boost environmental self-identity (*b* = 0.490, *t* = 10.759, and *p* < 0.001), which supports H3. The effect of pride feeling on costless saving behavior, however, is not significant (H4; *b* = 0.091, *t* = 1.534, and *p* = 0.125). Males have more costless saving behaviors than females (*b* = 0.167^∗∗∗^, *t* = 4.003, and *p* < 0.001), but there is no gender difference in costly saving behaviors (*b* = -0.081^∗∗∗^, *t* = 1.556, and *p* > 0.1). No matter in rural or urban areas, the growth background has no significant impact on saving behaviors (*b* = -0.075^∗∗∗^, *t* = 1.784, and *p* > 0.05).

The model explains 33.8 percent of the variance in environmental self-identity (adjusted *R*^2^ = 0.335), 15.7 percent of the variance in pride feeling (adjusted *R*^2^ = 0.155), and 28.6 percent of the variance in saving behavior (adjusted *R*^2^ = 0.280). The fit of the structural model is good, with a standard root mean-square residual = 0.040, which is lower than the benchmark of 0.05.

#### Costly Saving Behavior as a Dependent Variable

Taking the costly saving behavior as the dependent variable of the structural equation model. Surprisingly, all the results are consistent with the costless saving behavior model except for the H5. That is, environmental self-identity marginally significantly negatively affects costly saving behavior, reflecting an opposite licensing effect compared to the first model (*b* = -0.139,*t* = 1.846, and *p* < 0.1).

The model explains 34.0 percent of the variance in environmental self-identity (adjusted *R*^2^ = 0.337), 15.7 percent of the variance in pride feeling (adjusted *R*^2^ = 0.155), and 4.9 percent of the variance in resource consumption (adjusted *R*^2^ = 0.042). The fit of the structural model is good, with a standard root mean-square residual = 0.044 which is lower than the benchmark of 0.05.

### Testing Recycling Cost as a Moderator

We have built on the proposed Ma et al. (2019) conceptual model by introducing recycling cost variable. Further analysis will investigate the moderating effects of this variable has on pride feeling (H7) and environmental self-identity (H6).

#### Recycling Cost as a Moderator of Effect of Recycling Efforts on Pride Feeling

We tested the moderating effect of recycling cost on pride feeling. The interaction between recycling efforts and recycling cost has a negative effect on pride feeling (*b* = -0.061, *t* = -3.361, and *p* < 0.001), which is the opposite of hypothesis H7. This result demonstrates that recycling cost negatively moderates the relationship between recycling efforts and pride feeling. When the cost of recycling is higher, the positive relationship between recycling effort and pride feeling is weaker, that is, the recycling effort causes less pride feeling. This means that if the individuals realized that they have spent more time, physical and mental energy in recycling activities, they may feel very troublesome rather than feel priding. On the contrary, if they don’t feel any trouble at all, they will feel more proud.

#### Recycling Cost as a Moderator of Effect of Recycling Efforts on Environmental Self-Identity

**FIGURE 2:**
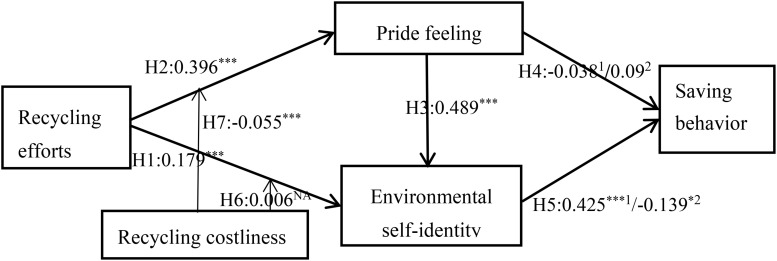
Results of hypotheses testing. Note.*p < 0.1 and ***p < 0.01. ^1^corrsponding to costless saving behavior, ^2^corrsponding to costly saving behavior. The figure synthesizes the path coefficients of two structural equation models with different dependent variables. Since the same sample data is used, the path coefficients corresponding to H1, H2, and H3 are the same, while the path coefficients corresponding to H4 and H5 are different.

We tested the moderating effect of recycling cost on environmental self-identity. The interaction between recycling efforts and recycling cost has no significant effect on environmental self-identity (*b* = -0.008, *t* = 0.179, and *p* = 0.858), which cannot support H6. This shows that the cost of recycling does not moderate the impact of recycling efforts on environmental self-identity. Whether the cost of recycling is high or low, recycling efforts have the same positive impact on environmental identity. This is not consistent with the original assumption, the possible reason is that the recycling cost is not actively accepted by the consumers, so it can not enhance environmental self-identity. Figure 2 provides summary results for all of the hypotheses.

## Discussion

This survey research provides evidence that individuals’ recycling efforts can increase their costless saving behaviors, while decrease their subsequent costly saving behaviors. We demonstrate that the efforts to recycle can influence subsequent resource saving by activating a positive pro-environmental self-identity, which gives individuals a license to take less costly saving behaviors, and at the same time, gives individuals a motive to take more costless saving behaviors. Research based on Construal Level Theory shows consumers save more when the saving goal is construed at a high level and tend to perceive specific goals as more difficult (Ülkümen and Cheema, 2011). Consumers usually don’t care about the costless saving behaviors in their daily life, which means that their psychological distances of these saving activities are distant, so they will construal these saving activities in a more abstract way. But for costly saving behaviors, such as those requiring some physical or mental effort, consumers will be more concerned with them and thus have a closer psychological distance, so they will construal them in a more concrete way. In one word, costliness is a key factor for individuals to make choices that are inconsistent or consistent with their prior recycling behaviors. In addition, we find that recycling cost negatively moderates the relationship between recycling efforts and pride feelings. It is worth noting that we conducted the same research on 224 college students in another university located far away, and the results still support this conclusion, which shows the robustness of the research conclusion.

### Contributions

The current study builds and extends the proposed conceptual model of recycling efforts and saving behavior proposed by Ma et al. (2019). The substantial changes introduced by the current study are the two-dimensional outcome of saving behavior and the moderating variable of recycling costs. The findings of this study therefore contribute to the existing literature in four ways.

First of all, we find that resource saving behavior is not a one-dimensional construct but can be divided into two types in terms of its costliness. As far as we know, there is no research to explore the nature of behavior toward resource saving or waste, neither to develop the measures to capture such behavior. Take an example, Ma et al. (2019) only takes the average monthly consumption (livelihood expenditure) as the measure of resource consumption level. The findings of this study provide directional guidance and verified measurement methods for investigating residents’ resource saving behavior.

Second, according to existing evidence (Ma et al., 2019), we assume that individuals who are highly involved in recycling activities will be less resource-efficient, that is, they will consume more resources. However, the research results show that recycling efforts promote costless saving behavior and inhibit costly saving behavior. For costly saving behaviors, individuals might feel allowed to relax moral requirements based on the moral licensing model (Mullen and Monin, 2016) due to the previous efforts on recycling activities. Therefore, their attitudes are more self-oriented in that they might have a negative appraisal of costly saving behaviors because they are unpleasant to them (Fishbein and Ajzen, 2010; Poškus, 2017). As a result, the behavior willingness toward costly saving behaviors is declined. On the contrary, costless behaviors are easy to implement, as well as comply with social norms, which resulted in a positive appraisal and stronger intentions to take. This finding reconciles existing studies on the conflicting conclusion on moral spillover effects in environmental behaviors. For example, Thøgersen (1999) and Thøgersen and Noblet (2012) suggested the positive spillover effects, i.e., continuation to engage in recycling after initial recycling activities. Other studies, however, pointed toward the negative spillover effects (Catlin and Wang, 2013; Nilsson et al., 2017; Ma et al., 2019). The results of this study explore the spillover effect of recycling and saving behavior and thus enrich the understanding of individual sustainable behavior dynamics.

Third, this study examines the impact path of recycling efforts on saving behavior. We find that both positive and negative spillovers are mediated by pride feeling and environmental self-identity, which is consistent with Ma et al. (2019). Furthermore, the pro-environmental identity can be, in fact, boosted by recycling behavior. This identity thus can result in greater engagement of costless recycling at the same time decreasing the engagement in costly saving behaviors. The opposing view of the moral licensing effect on recycling behavior (Catlin and Wang, 2013; Sun and Trudel, 2017) are more in line with the current study findings and further explores the psychological mechanism of the effect. At the same time, this study has taken a great step forward on the basis of Ma et al. (2019). Their research adopted average monthly consumption (livelihood expenditure) as the proxy variable of resource consumption and finds the positive spillover effect of recycling efforts on resource consumption. In this study, six common resource saving behaviors of college students were taken as dependent variables and revealed the divergent effects of recycling efforts on differential saving behaviors.

Forth, this study finds evidence for the moderating effect of recycling cost between recycling efforts and pride feeling. However, the direction of moderation is just opposite to our prediction, i.e., when the cost of recycling is higher, the pride of recycling effort is lower than that of lower recycling cost. This result is also inconsistent with Sun and Trudel (2017), in whose utilitarian model it is believed that pro-environmental behaviors with high costs will bring stronger positive emotions. We have carefully examined the research data to confirm that the research results are reliable. Furthermore, we interviewed some of the respondents to understand why such unexpected results occurred. Finally, we think that an important feature of our college students’ sample leads to this result, that is, they do not always take the initiative to recycle resources in their daily life, or even feel very troublesome in many situations. Because there are many social norms in China’s powerful collectivism culture, even if many people are not willing to spend a lot of efforts to recycle wastes, they still have to do recycling under the social pressure. As a result, we argue that whether recycling costs enhance or weaken the positive emotions from recycling efforts depends on whether individuals actively or passively accept the recycling costs. The respondents in this study might have to implement costly recycling behavior under social pressure. Therefore, they felt troubles, reluctance and other negative emotions, which offset feeling of pride. Take the same example from Sun and Trudel (2017), putting the recycling bin farther away might produce stronger positive emotions for some people who has a society-oriented attitude, but might produce stronger negative emotions for one who who has a self-oriented attitude, therefore dislikes walking so far to recycle. For the same reason, costly recycling behavior is not able to diagnostic about oneself (Burger, 1999; Gneezy et al., 2012). Because recycling isn’t an inner initiative, individuals who passively accept the higher cost of recycling cannot prove that they are a pro-environmental person, thus can not strengthening their environmental self-identity. As a result, there is no significant moderating effect of recycling cost between recycling efforts and environmental self-identity. Anywhere, this is a problem worthy of further study.

### Management Implications

The above research reveals that many people want to improve their environmental identity, but are unwilling to pay the price. People are willing to take easy-to-implement environmentally sustainable behaviors in order to get a good sense of self. However, when there is a price to pay, past pro-environmental behavior such as recycling efforts may be used as a reason for self-excuse. According to the economic theory, it is human nature to maximize gains and to minimize loses (Camerer, 1997). Therefore, it is unrealistic to insist for people to be selfless and pay personal costs for the environment.

The increased use of modern technologies by government could have impacts on increasing saving behavior and decrease costs of recycling. Although high recycling cost can directly reduce current consumption, it also brings a lot of trouble to daily life. For example, in July 2019 in Shanghai, China, the first implementation of the compulsory classification policy of domestic waste greatly constrains the consumption behavior of residents. In order to avoid annoying garbage classification, many people reduce the frequency of taking-out food ordered online, or abandon some food that is difficult to classify, such as pearl milk tea. Although this policy can promote consumption reduction, it can also lead to negative emotions, which may reduce the pride feeling and environmental identity brought by recycling behavior, and then may inhibit some low-cost saving behaviors, such as saving water and electricity when staying in hotels. In order to reduce the cost of classified recycling, the government can increase training guidance on how to classify garbage and support the establishment of professional recycling intermediaries. In addition, the government should invest more in the recycling infrastructure and make it easier to recycle by using intelligent and internet-based means, such as making it easy for people to find the recycling sites of toxic waste through a mobile app.

On the other hand, the policy measures to reduce the costliness of saving can start with publicizing and popularizing the knowledge of saving, such as telling people the tips of saving resources through celebrities or web-casters, which is more persuasive. In addition, guiding people to establish the value of “I am thrifty and proud” and advocating “minimalist lifestyle” can reduce the psychological cost of some economizing behaviors, such as wearing old clothes and shoes.

### Limitations and Directions for Further Research

This study provides insights into recycling and resource saving, although it inevitably has some limitations that can be addressed through future research. First, the self-report data by nature pose certain issues. We encourage future research to explore further relevant research issues using field studies or big data. Second, for understandable and common reasons, the object of this study is college students, and the conclusions should not be extended to other groups before being verified. Third, the measure of saving behavior is still a preliminary exploratory work, and it is valuable to improve it in the future. Forth, we cannot assess all the moderating variables between recycling behaviors and resource saving (e.g., social pressure, environmental values), which is a promising way for further research. Finally, since the research is carried out in the Chinese cultural context, while the conclusions obtained are instructive for the Chinese context, we need to be vigilant about the adaptability of the conclusions in any other cultural context.

## Data Availability Statement

The original contributions presented in the study are included in the article/supplementary material, further inquiries can be directed to the corresponding author/s.

## Ethics Statement

The studies involving human participants were reviewed and approved by Academic Committee of Center for Behavior and Decision Shaanxi University of Technology. Written informed consent for participation was not required for this study in accordance with the national legislation and the institutional requirements.

## Author Contributions

SS and SW: conceptualization. YW: data curation. JX: formal analysis. SW and SS: investigation. JX: methodology. SS: project administration. SS and YZ: supervision. YW: writing – original draft. YZ: writing – review and editing. All authors contributed to the article and approved the submitted version.

## Conflict of Interest

The authors declare that the research was conducted in the absence of any commercial or financial relationships that could be construed as a potential conflict of interest.

## Footnotes

1 https://www.epa.gov/recycle/recycling-basics
